# Supplementary material for: Impact of parental education on number of under five children death per mother in Bangladesh
Source: PLoS One. 2025 Feb 10;20(2):e0318787. doi: 10.1371/journal.pone.0318787 (PMC11809813; doi:10.1371/journal.pone.0318787)

PLOS ONE

Impact of parental education on number of under five children death per mother in Bangladesh

--Manuscript Draft--

**Manuscript Number:**	PONE-D-24-22195
**Article Type:**	Research Article
**Full Title:**	Impact of parental education on number of under five children death per mother in Bangladesh
**Short Title:**	Impact of parental education on number of under five children death
**Corresponding Author:**	Bikash Pal, MS<br>University of Dhaka<br>Dhaka, BANGLADESH
**Keywords:**	parental education; children death; zero inflated negative binomial model
**Abstract:**	One of the leading challenges of social development is the reduction of children's death at age under five. The primary focus of this research is to study the potential impact of parental education on under five children death in Bangladesh. The total count of deceased children within a family is a non-negative numerical variable. The mean number of under five children death per 100 mother is found to be 20 with variance around 27, which indicates the presence of overdispersion. As the response variable exhibits 84.2% zero counts, we have considered three regression models in this research; Poisson model, zero-inflated Poisson model, and zero-inflated negative binomial model. Finally, zero-inflated negative binomial model, exhibiting the lowest AIC value, indicates that both maternal and paternal education have significant protective impact on under five children death. Specifically, greater levels of formal education achieved by the parents are associated with a decreased rate of children death.
**Order of Authors:**	Farzana Afroz, Ph.D.
	Md. Muddasir Hossain Akib, MS
	Bikash Pal, MS
	Abida Sultana Asha, MS
**Additional Information:**	
**Question**	**Response**
**Financial Disclosure**<br><br>Enter a financial disclosure statement that describes the sources of funding for the work included in this submission. Review the submission guidelines for detailed requirements. View published research articles from *PLOS ONE* for specific examples.<br><br>This statement is required for submission and **will appear in the published article** if the submission is accepted. Please make sure it is accurate.	The author(s) received no specific funding for this work.

**Funded studies**<br>Enter a statement with the following details:<br>• Initials of the authors who received each award<br>• Grant numbers awarded to each author<br>• The full name of each funder<br>• URL of each funder website<br>• Did the sponsors or funders play any role in the study design, data collection and analysis, decision to publish, or preparation of the manuscript?<br><br>Did you receive funding for this work?	
**Competing Interests**<br><br>Use the instructions below to enter a competing interest statement for this submission. On behalf of all authors, disclose any competing interests that could be perceived to bias this work—acknowledging all financial support and any other relevant financial or non-financial competing interests.<br><br>This statement is required for submission and **will appear in the published article** if the submission is accepted. Please make sure it is accurate and that any funding sources listed in your Funding Information later in the submission form are also declared in your Financial Disclosure statement.<br><br>View published research articles from *PLOS ONE* for specific examples.	The authors have declared that no competing interests exist.

Powered by Editorial Manager® and ProduXion Manager® from Aries Systems Corporation

**NO authors have competing interests**<br><br>Enter: *The authors have declared that no competing interests exist.*<br><br>**Authors with competing interests**<br><br>Enter competing interest details beginning with this statement:<br><br>*I have read the journal's policy and the authors of this manuscript have the following competing interests: [insert competing interests here]*<br><br>* typeset	
**Ethics Statement**<br><br>Enter an ethics statement for this submission. This statement is required if the study involved:<br><br>• Human participants<br>• Human specimens or tissue<br>• Vertebrate animals or cephalopods<br>• Vertebrate embryos or tissues<br>• Field research<br><br>Write "N/A" if the submission does not require an ethics statement.<br><br>General guidance is provided below. Consult the submission guidelines for detailed instructions. **Make sure that all information entered here is included in the Methods section of the manuscript.**	The Institutional Review Board of ICF International, Rockville, Maryland, USA (formerly Macro International) reviewed and approved the Demographic and Health Surveys (DHS) Program, including the 2017–18 Bangladesh DHS. Additionally, the 2017–18 Bangladesh DHS received approval from the Bangladesh Medical Research Council. This survey was conducted by the National Institute of Population Research and Training (NIPORT) under the authority of the Government of the People's Republic of Bangladesh, with financial support from USAID/Bangladesh. Informed consent was obtained from each survey participant prior to their involvement, and those who did not consent were not included in the survey.

Powered by Editorial Manager® and ProduXion Manager® from Aries Systems Corporation

**Format for specific study types**<br><br>**Human Subject Research (involving human participants and/or tissue)**<br>• Give the name of the institutional review board or ethics committee that approved the study<br>• Include the approval number and/or a statement indicating approval of this research<br>• Indicate the form of consent obtained (written/oral) or the reason that consent was not obtained (e.g. the data were analyzed anonymously)<br><br>**Animal Research (involving vertebrate animals, embryos or tissues)**<br>• Provide the name of the Institutional Animal Care and Use Committee (IACUC) or other relevant ethics board that reviewed the study protocol, and indicate whether they approved this research or granted a formal waiver of ethical approval<br>• Include an approval number if one was obtained<br>• If the study involved *non-human primates*, add *additional details* about animal welfare and steps taken to ameliorate suffering<br>• If anesthesia, euthanasia, or any kind of animal sacrifice is part of the study, include briefly which substances and/or methods were applied<br><br>**Field Research**<br><br>Include the following details if this study involves the collection of plant, animal, or other materials from a natural setting:<br>• Field permit number<br>• Name of the institution or relevant body that granted permission	
**Data Availability**<br><br>Authors are required to make all data underlying the findings described fully available, without restriction, and from the time of publication. PLOS allows rare exceptions to address legal and ethical concerns. See the PLOS Data Policy and FAQ for detailed information.	Yes - all data are fully available without restriction

A Data Availability Statement describing where the data can be found is required at submission. Your answers to this question constitute the Data Availability Statement and **will be published in the article**, if accepted.<br><br>**Important:** Stating 'data available on request from the author' is not sufficient. If your data are only available upon request, select 'No' for the first question and explain your exceptional situation in the text box.<br><br>Do the authors confirm that all data underlying the findings described in their manuscript are fully available without restriction?	
**Describe where the data may be found in full sentences. If you are copying our sample text, replace any instances of XXX with the appropriate details.**<br><br>• If the data are **held or will be held in a public repository**, include URLs, accession numbers or DOIs. If this information will only be available after acceptance, indicate this by ticking the box below. For example: *All XXX files are available from the XXX database (accession number(s) XXX, XXX.)*.<br>• If the data are all contained **within the manuscript and/or Supporting Information files**, enter the following: *All relevant data are within the manuscript and its Supporting Information files.*<br>• If neither of these applies but you are able to provide **details of access elsewhere**, with or without limitations, please do so. For example:<br><br>*Data cannot be shared publicly because of [XXX]. Data are available from the XXX Institutional Data Access / Ethics Committee (contact via XXX) for researchers who meet the criteria for access to confidential data.*<br><br>*The data underlying the results presented in the study are available from (include the name of the third party*	The dataset utilized for this study is accessible in the following DHS repository https://dhsprogram.com/data/available-datasets.cfm.

and contact information or URL).<br>• This text is appropriate if the data are owned by a third party and authors do not have permission to share the data.<br><br>* typeset	
Additional data availability information:	

Powered by Editorial Manager® and ProduXion Manager® from Aries Systems Corporation

Manuscript
Click here to access/download;Manuscript;Manuscript 16-05-24.docx

Impact of Parental Education on Number of Under Five Children Death Per Mother in Bangladesh

Farzana Afroz[1], Md. Muddasir Hossain Akib[1], Bikash Pal[1*], Abida Sultana Asha[1]

[1]Department of Statistics, University of Dhaka, Dhaka- 1000, Bangladesh

[*]**Corresponding author:** Bikash Pal, Department of Statistics, University of Dhaka, Dhaka- 1000, Bangladesh**,** e-mail: bikashpal@du.ac.bd

All the authors contributed equally to this work.

Abstract

One of the leading challenges of social development is the reduction of children's death at age under five. The primary focus of this research is to study the potential impact of parental education on under five children death in Bangladesh. The total count of deceased children within a family is a non-negative numerical variable. The mean number of under five children death per 100 mother is found to be 20 with variance around 27, which indicates the presence of overdispersion. As the response variable exhibits 84.2% zero counts, we have considered three regression models in this research; Poisson model, zero-inflated Poisson model, and zero-inflated negative binomial model. Finally, zero-inflated negative binomial model, exhibiting the lowest AIC value, indicates that both maternal and paternal education have significant protective impact on under five children death. Specifically, greater levels of formal education achieved by the parents are associated with a decreased rate of children death.

Introduction

Child mortality stands as a pivotal measure of a nation's prosperity and societal advancement, echoing loudly in the global discourse on public health. The under five child mortality rate, representing the risk of a child perishing before reaching the age of five, serves as a critical gauge of socioeconomic development and living standards. The Sustainable Development Goals (SDGs) aim for a significant reduction in neonatal and under-five mortality rates by 2030, targeting 12 and 25 deaths per 1,000 live births, respectively (1). Currently, 79 nations grapple with under five mortality rates exceeding 25 per 1000 live births (2). The case of Bangladesh is noteworthy, as under-five mortality rates witnessed a substantial decrease. Specifically, between 1990 and 2018, there was a significant drop in mortality rates, credited to the effective implementation of the Millennium Development Goals (MDGs) (3). As Bangladesh strives to align with SDG 3, aiming to reduce mortality for newborns and children under five to 25 per 1000 live births by 2030 (4), a critical examination of the strategies employed becomes imperative.

This paper scrutinizes parental education as a key determinant of child mortality reduction in Bangladesh. Drawing on nationwide survey data, our study underscores the pivotal role of primary caregivers' education as a potential pathway for Bangladesh to achieve SDGs. While various studies have explored socioeconomic and demographic factors influencing child mortality in Bangladesh, only a limited number have delved into the specific impact of parental education. Addressing this research gap, our study specifically underscores the impact of paternal education on modelling mortality of children in Bangladesh. Despite beliefs in the contribution of both parents' education to child health, existing studies present mixed findings (5-9). For instance, research in Indonesia suggests that both maternal and paternal education exhibit a similar association with under five mortality (8), while others indicate a weaker or insignificant link between paternal education and death of children compared to maternal education (5, 6, 10).

Increasing evidence emphasizes the protective effect of maternal education on child mortality (11, 12). Mothers who have attained advanced education levels typically exhibit greater familiarity with services related to maternal health, leading to enhanced outcomes during childbirth and the postnatal period. Moreover, their awareness of advancements in healthcare facilitates access to essential services for both themselves and their newborns. Maternal education also equips mothers with the knowledge to identify health discrepancies in their children early on, fostering timely interventions.

While the effect of paternal education on child mortality remains debated, Akter et al. (13) discovered that higher maternal education is correlated with a 38% reduction in under five mortality, whereas greater paternal education contributed a 16% reduction. In the socio-cultural context of Bangladesh, where fathers are often considered household heads responsible for financial matters, their lack of formal education may hinder their awareness of necessary healthcare for their offspring. This knowledge gap might lead them to opt for cost-effective alternatives, such as traditional village doctors, potentially jeopardizing newborn health.

In line with this backdrop, our research examines the link between the parental education level and the under five mortality rates in Bangladesh. Additionally, we consider other covariates to account for potential confounding effects of parental education. Various regression models have been explored in existing literature to analyse count responses, including the Poisson Regression model (PRM), Negative Binomial Regression model (NBRM), and Generalized Poisson Regression model (GPRM) (14). The application of PRM is contingent on the assumption that the value of the mean and the variance of the response variable are equal. The GPRM stands out as a versatile tool adept at handling both overdispersion or underdispersion, while the NBRM is specifically recommended for addressing overdispersion (15, 16). However, the presence of excess count of zeros in the response variable necessitates a nuanced approach. In this study, we opt for the Poisson Regression Model, the zero-Inflated Poisson Regression model, and the zero-inflated negative binomial regression (15) to effectively model the number of children's deaths per mother within the regression framework.

Our study seeks to enhance the existing literature by clarifying trends in the relationship between parental education and under five mortality. On the policy front, our findings may inform strategies for combating child mortality by shedding light on the evolving significance of parental education.

Methods

Data and variables

This study utilizes a dataset sourced from the 2017-18 Bangladesh Demographic and Health Survey (BDHS). This survey, part of a series of national-level population and health surveys, is designed to provide comprehensive data on the demographic and health conditions of women and children in Bangladesh (17).

This study centers on child mortality rates per mother in Bangladesh, focusing specifically on children under the age of five. The data utilized originates from the BDHS 2017-18, which collected information from 20,127 ever-married women aged 15-49 on 5,243 variables. Among all the

individuals, data from women who have not given any birth have been considered irrelevant with the title and objective of the study, and hence discarded. And lastly, 18134 women have been considered here, who have ever given birth to any child.

In this paper, the number of children's death per mother (CDPM) has been derived from the variable-age at death (months imputed), where mothers having children dying before age 5 years (60 months) have been taken into account and it resulted in a count variable. Therefore, the count response variable of our interest is the number of under five children death for each mother.

As the explanatory variables, a total of nine covariates have been considered here, namely, administrative division (region), type of place of residence, mother's education level, father's education level, wealth index, birth status, exposure to media and mother's working status. As some data on specific variables were unavailable directly from the 2017-18 BDHS dataset, we adjusted their categorization for analysis. For instance, to define the "exposed to media" variable, we grouped mothers based on their regular engagement with activities such as reading newspapers or magazines, listening to the radio, or watching television on a weekly basis (18). In the variable birth status, a mother who has given birth to two or more children has been categorized as multiple, while a mother with only one child belongs to the category of single.

Ethics approval and consent to participate

The Institutional Review Board of ICF International, Rockville, Maryland, USA (formerly Macro International) reviewed and approved the Demographic and Health Surveys (DHS) Program, including the 2017–18 Bangladesh DHS. Additionally, the 2017–18 Bangladesh DHS received approval from the Bangladesh Medical Research Council. This survey was conducted by the National Institute of Population Research and Training (NIPORT) under the authority of the Government of the People's Republic of Bangladesh, with financial support from USAID/Bangladesh. Informed

consent was obtained from each survey participant prior to their involvement, and those who did not consent were not included in the survey.

Statistical analysis

For analysing under five children death count data, we considered three regression models Poisson regression model, zero-inflated Poisson model and zero-inflated negative binomial model, in the context of generalized modelling framework (19). The basic Poisson regression model with equidispersion assumption can be used for analysing count data, however in most of the cases the data are overdispersed. When overdispersion is present, negative binomial distribution is preferred which allows Poisson mean to vary following gamma distribution. In data with excess zero count the zeros may not arise by the same mechanism as the other count part (20). To put up with the extra zeros observed in the data, zero inflated Poisson (ZIP) model is proposed (21). In zero inflated data the mean and variance may not be equal, to accommodate both zero inflation and overdispersion a natural choice of the model can be the zero inflated negative binomial distribution (22).

When the count response (Y_i) follows a Poisson distribution, the zero -inflated Poisson (ZIP) model can be written as

$$P(Y_i = y_i) = \begin{cases} p_i + (1-p_i)e^{-\mu_i} & if\ y_i = 0, \\ (1-p_i)\frac{e^{-\mu_i}{\mu_i}^{y_i}}{y_i!} & if\ \ y_i > 0 \end{cases} ,$$

where, p_i is the probability of a structural zero. A Poisson (μ_i) random variable is observed in Y_i with probability $1 - p_i$ and μ_i is the mean of the standard Poisson distribution. Mean and variance of ZIP model are $E(Y_i) = (1-p_i)\mu_i$ and $V(Y_i) = (1-p_i)(\mu_i + \mu_i^2) - ((1-p_i)\mu_i)^2$, respectively. Again, the zero-inflated negative binomial (ZINB) model is expressed as follows

$$P(Y_i = y_i) = \begin{cases} p_i + (1-p_i)\left[\left(\frac{\phi}{\mu_i+\phi}\right)^{\phi}\right] & if\ y_i = 0 \\ (1-p_i)\frac{\Gamma(y_i+\phi)}{y_i!\Gamma(\phi)}\left(\frac{\mu_i}{\mu_i+\phi}\right)^{y_i}\left(\frac{\phi}{\mu_i+\phi}\right)^{\phi} & if\ \ y_i > 0 \end{cases} ,$$

where ϕ indicates the over dispersion parameter. Mean and variance of ZINB model are $E(Y_i) = (1 - p_i)\mu_i$ and $V(Y_i) = (1 - p_i)\mu_i\ (1 - p_i\mu_i + \mu_i/\phi)$, respectively. The model with the smallest AIC [27] is used to analyze the data. For a model with p parameters, AIC can be presented as, $\text{AIC} = -2l(\widehat{\boldsymbol{\theta}}, \boldsymbol{y}) + 2p$, where $l(\widehat{\boldsymbol{\theta}}, y)$ represents the log-likelihood. For the purpose of exploring the effects of the covariates on the count data incidence rate ratio (IRR) is used, which can be written as: $IRR_j = \exp(\hat{\beta}_j)$, where $\hat{\beta}_j$ represents the estimated regression coefficient for the j-th covariate.

Results

The histogram presented in Fig 1 indicates that the occurrence of more than 2 child deaths is extremely rare. Additionally, it highlights that a significant majority of mothers (84.21%) experienced no child death. Since the frequency for zero in count of children death per mother is extremely high, we call this a zero inflated count data.

Fig 1. Histogram for the number of under five children death per mother.

The existence of overdispersion in the dataset becomes apparent, as indicated by Table 1, where the variance of the number of under five children deaths per 100 mothers (27) significantly exceeds the mean number of under-five children deaths per 100 mothers (20).

Table 1. Descriptive statistics for the number of under five children death per 100 mothers.

Characteristics	CDPM
Size of the Sample (Total number of respondents)	18134
Sample Mean	20
Sample Variance	27

Therefore, it is easily understood that apart from the presence of too many zeros in the underlying dataset, we need to deal with the presence of overdispersion at the same time. Our study employs three regression models; the Poisson Regression Model, zero-Inflated Poisson Regression model, and zero-inflated negative binomial regression. To determine the optimal model fit for our data, our study compares the models using the Akaike Information Criteria (AIC).

As shown in Table 2, the zero-inflated negative binomial exhibits the lowest AIC value (15371.97) among all three models. Thus, we will focus on the resulting values obtained from fitting the zero-inflated negative binomial regression model. Our study reveals that the determinants region, mother's education level, father's education level, mother's age at 1st birth, wealth index, and birth status significantly affect the number of children's deaths per mother in Bangladesh. The mean number of deaths of children per mother in the Chittagong division is significantly (p-value = 0.041, IRR = 0.851) 14.9% lower than that of the Dhaka division. Residence type has been found to be insignificant. All the categories included in the mother's educational level have been found to be significant. The mean numbers of deaths of children for mothers with primary, secondary, and higher levels of education are respectively 22%, 33.4%, and 39.9% lower in comparison to the number of deaths of children for mothers with no education. Similar outcomes have also been observed regarding the educational levels of fathers. The average numbers of deaths of children per mother are respectively 8.4%, 17.5%, and 20% lower for fathers with primary, secondary, and higher levels of education in comparison to the number of deaths of children per mother married to fathers with no education. Statistically significant results were obtained for mothers aged 18-25 at the time of their first birth (p-value <0.001), with the mean number of child deaths per mother being 16.7% lower than that of mothers who were younger than 18 at the time of their first birth. However, the working status of mothers and their exposure to media did not show significant impacts on the number of child deaths. From the reported p-values, we can see that the wealth index has a significant effect on the number of children's deaths per mother as the women from rich families have a 13.1% lower average number of deaths of children per mother compared to the women belonging to poor families. Finally, the explanatory variable birth status is statistically significant (p-value <0.001), and an IRR value of 7.441 implies that the average number of deaths of children per mother in case of multiple birth status is 644.1% higher than that of single and double birth status.

Table 2. Results obtained from different regression models along with their respective AIC values.

	Poisson regression		Zero-Inflated Poisson		Zero-Inflated negative binomial	
**Covariates**	**IRR**	**p-value**	**IRR**	**p-value**	**IRR**	**p-value**
**Intercept**	0.065	**<0.001**	0.076	**<0.001**	0.082	**<0.001**
**Region**						
Dhaka	-	-	-	-	-	-
Barisal	1.006	0.925	0.904	0.477	0.938	0.462
Chittagong	0.916	0.200	0.815	**0.099**	0.851	**0.041**
Khulna	0.932	0.340	0.630	**0.001**	0.984	0.852
Mymensingh	1.053	0.463	0.887	0.302	0.968	0.704
Rajshahi	1.089	0.213	0.822	0.155	1.030	0.719
Rangpur	0.970	0.667	0.784	**0.054**	0.987	0.881
Sylhet	1.142	**0.051**	1.083	0.508	1.077	0.346
**Place Type**						
Rural	-	-	-	-	-	-
Urban	1.032	0.432	1.094	0.151	0.989	0.832
**Respondent's Education Level**						
No education	-	-	-	-	-	-
Primary	0.764	**<0.001**	0.724	**<0.001**	0.780	**<0.001**
Secondary	0.657	**<0.001**	0.648	**<0.001**	0.666	**<0.001**
Higher	0.512	**<0.001**	0.365	**<0.001**	0.601	**0.001**
**Partner's Education Level**						
No education	-	-	-	-	-	-
Primary	1.001	0.969	1.076	0.330	0.916	**0.098**
Secondary	0.888	**0.028**	0.997	0.969	0.825	**0.003**
Higher	0.856	**0.088**	0.923	0.627	0.800	**0.032**
**Respondent's Age at 1st Birth**						
<18	-	-	-	-	-	-
18-25	0.880	**<0.001**	0.957	0.065	0.833	**<0.001**
>25	1.037	0.798	1.500	**<0.001**	0.910	0.563
**Respondent's Working Status**						
No	-	-	-	-	-	-
Yes	1.016	0.658	0.938	0.247	1.033	0.441
**Media Exposure**						
Not Exposed	-	-	-	-	-	-
Exposed	0.968	0.433	0.939	0.369	0.974	0.590
**Wealth Index**						
Poor	-	-	-	-	-	-
Middle	0.979	0.626	1.099	0.213	0.999	0.986
Rich	0.845	**0.015**	0.842	0.103	0.869	**0.078**
**Birth Status**						

Single/double	-	-	-	-	-	-
Multiple	8.348	**<0.001**	10.309	**<0.001**	7.441	**<0.001**
**AIC**	15472.00		15402.43		15371.97	

Discussion

The inferential findings outlined in this research emphasize the significant impact of parental education on shaping child mortality in Bangladesh. The results suggest a clear association between elevated levels of parental education and a reduction in the average number of child deaths per mother, consistent with earlier research on this subject (5, 23). The discussion emphasizes the multifaceted benefits of maternal education, ranging from increased awareness about health and nutrition to improved decision-making abilities and greater knowledge about disease prevention. These factors collectively contribute to a more favourable environment for child health and well-being. The shift towards educated females participating in the workforce has led to a positive change in societal norms, enabling women to voice their opinions and make informed decisions. Additionally, the research highlights the beneficial influence of education on men, observing that fathers with higher education levels are inclined towards assisting women in making reproductive choices and healthcare decisions.

While education emerges as a significant factor, the study acknowledges the influence of other sociodemographic factors on child mortality. Maternal age at first birth emerges as a significant contributor, with a consistent trend showing a decrease in the mean number of child deaths as the age of the mother increases. This aligns with existing research, suggesting that teenage mothers may lack awareness of maternal care, potentially contributing to higher child mortality rates (24). Financial status proves to be a crucial factor, as mothers from affluent families exhibit lower number of child deaths compared to their economically disadvantaged counterparts. This finding resonates with prior studies and emphasizes the intricate link between financial resources and the availability of healthcare services for mothers and children (25, 26). The study underscores the challenges faced by

impoverished parents, who may struggle to afford even basic needs, let alone essential healthcare services. Additionally, the study sheds light on the impact of birth status, revealing a strong association between multiple births and increased child mortality rates. This observation aligns with conclusions drawn from similar investigations (27, 28). The challenges associated with managing the health and well-being of multiple children simultaneously likely contribute to this phenomenon. Furthermore, regional disparities emerge as a notable factor influencing child mortality rates. The result aligns with previous research by identifying variations in the number of child deaths per mother across different regions (29, 30). This regional dimension emphasizes the importance of tailoring healthcare policies and interventions to address specific challenges faced by communities in different geographical areas.

Conclusion

In delineating the intricacies of child mortality in Bangladesh, this study illuminates the indispensable role of parental education as a potent factor in reduction. The application of a zero-inflated negative binomial regression model, considering zero-inflated count data and overdispersion, revealed significant determinants such as region, parental education levels, maternal age at first birth, wealth index, and birth status. The study emphasizes the need for targeted interventions addressing socioeconomic factors, maternal age, and regional variations to comprehensively improve child health outcomes. To enhance child survival, policy initiatives should prioritize parental education, and address the specific challenges faced by different regions and socioeconomic groups.

Contributors

FA: Conceptualization; Methodology; Formal Analysis; Writing - Original Draft. MHA: Conceptualization; Writing - Original Draft. BP: Writing - Original Draft; Visualization. ASA: Conceptualization, Data Curation. All authors: Review & Editing.

Data availability

The dataset utilized for this study is accessible in the following DHS repository https://dhsprogram.com/data/available-datasets.cfm.

Ethical statement

As we utilized secondary data from the DHS for our study, ethical approval was not deemed necessary. However, DHS Program authority granted us to download and use the data files.

Acknowledgment

We express our appreciation to the DHS (Demographic and Health Surveys) for granting us access to their dataset for our research. Furthermore, we acknowledge the National Institute of Population Research and Training (NIPORT) for conducting the BDHS, 2017–2018.

References

1. Chao F, You D, Pedersen J, Hug L, Alkema L. National and regional under-5 mortality rate by economic status for low-income and middle-income countries: a systematic assessment. The Lancet Global Health. 2018;6(5):e535-e47.

2. Bustreo F, Okwo-Bele J, Kamara L. World Health Organization perspectives on the contribution of the Global Alliance for Vaccines and Immunization on reducing child mortality. Archives of disease in childhood. 2015;100(Suppl 1):S34-S7.

3. Rugg D, Marais H, Carael M, De Lay P, Warner-Smith M. Are we on course for reporting on the Millennium Development Goals in 2015? JAIDS Journal of Acquired Immune Deficiency Syndromes. 2009;52:S69-S76.

4. UNICEF. Progress for every child in the SDG ERA are we on track to achieve the SDGs for children? the situation in 2019. 2019.

5. Caldwell JC. Education as a factor in mortality decline an examination of Nigerian data. Population studies. 1979:395-413.

6. Cochrane SH, Leslie J, O'Hara DJ. Parental education and child health: intracountry evidence. Health policy and education. 1982;2(3-4):213-50.

7. Kiros G-E, Hogan DP. War, famine and excess child mortality in Africa: the role of parental education. International journal of epidemiology. 2001;30(3):447-55.

8. Breierova L, Duflo E. The impact of education on fertility and child mortality: Do fathers really matter less than mothers? : National bureau of economic research Cambridge, Mass., USA; 2004.

9. Macassa G, Ghilagaber G, Bernhardt E, Diderichsen F, Burström B. Inequalities in child mortality in Mozambique: differentials by parental socio-economic position. Social science & medicine. 2003;57(12):2255-64.

10. Khanal V, Gavidia T, Adhikari M, Mishra SR, Karkee R. Poor thermal care practices among home births in Nepal: further analysis of Nepal Demographic and Health Survey 2011. PloS one. 2014;9(2):e89950.

11. Abir T, Agho KE, Page AN, Milton AH, Dibley MJ. Risk factors for under-5 mortality: evidence from Bangladesh Demographic and Health Survey, 2004–2011. BMJ open. 2015;5(8):e006722.

12. Khan JR, Awan N. A comprehensive analysis on child mortality and its determinants in Bangladesh using frailty models. Archives of Public Health. 2017;75:1-10.

13. Akter T, Hoque D, Chowdhury E, Rahman M, Russell M, Arifeen S. Is there any association between parental education and child mortality? A study in a rural area of Bangladesh. Public health. 2015;129(12):1602-9.

14. Islam MM, Alam M, Tariquzaman M, Kabir MA, Pervin R, Begum M, et al. Predictors of the number of under-five malnourished children in Bangladesh: application of the generalized poisson regression model. BMC public health. 2013;13:1-8.

15. Alam M, Farazi MR, Stiglitz J, Begum M. Statistical modeling of the number of deaths of children in Bangladesh. Biometrics & Biostatistics International Journal. 2014;1.

16. Akib MMH, Pal B. Analyzing Overdispersed Skilled Antenatal Care Visits of Pregnant Women in Bangladesh Using Generalized Poisson Regression Model.

17. National Institute of Population Research and Training (NIPORT), and ICF., 2020. Bangladesh Demographic and Health Survey 2017-18. Dhaka, Bangladesh, and Rockville, Maryland, USA: NIPORT and ICF.

18. Sultana N, Bari W. Analyzing Overdispersed Antenatal Care Visits of Pregnant Women in Bangladesh: Negative Binomial Regression Model. Dhaka University Journal of Science. 2017;65(2):133-7.

19. McCullagh P. Generalized linear models: Routledge; 2019.

20. Lambert D. Zero-inflated Poisson regression, with an application to defects in manufacturing. Technometrics. 1992;34(1):1-14.

21. Greene WH. Accounting for excess zeros and sample selection in Poisson and negative binomial regression models. 1994.

22. Mwalili SM, Lesaffre E, Declerck D. The zero-inflated negative binomial regression model with correction for misclassification: an example in caries research. Statistical methods in medical research. 2008;17(2):123-39.

23. Cleland JG, Van Ginneken JK. Maternal education and child survival in developing countries: the search for pathways of influence. Social science & medicine. 1988;27(12):1357-68.

24. Chowdhury AH. Determinants of Under-Five Mortality in Bangladesh. Open Journal of Statistics. 2013;03(03):213-9.

25. Asif MF, Pervaiz Z, Afridi JR, Safdar R, Abid G, Lassi ZS. Socio-economic determinants of child mortality in Pakistan and the moderating role of household’s wealth index. BMC pediatrics. 2022;22:1-8.

26. Van Malderen C, Van Oyen H, Speybroeck N. Contributing determinants of overall and wealth-related inequality in under-5 mortality in 13 African countries. J Epidemiol Community Health. 2013;67(8):667-76.

27. Yaya S, Bishwajit G, Okonofua F, Uthman OA. Under five mortality patterns and associated maternal risk factors in sub-Saharan Africa: a multi-country analysis. PloS one. 2018;13(10):e0205977.

28. Kembo J, Van Ginneken JK. Determinants of infant and child mortality in Zimbabwe: Results of multivariate hazard analysis. Demographic Research. 2009;21:367-84.

29. Antai D. Regional inequalities in under-5 mortality in Nigeria: a population-based analysis of individual-and community-level determinants. Population health metrics. 2011;9:1-10.

30. Feng XL, Guo S, Yang Q, Xu L, Zhu J, Guo Y. Regional disparities in child mortality within China 1996–2004: epidemiological profile and health care coverage. Environmental health and preventive medicine. 2011;16:209-16.

Click here to access/download;Figure;Fig 1.docx

Fig 1. Histogram for the number of under five children death per mother.

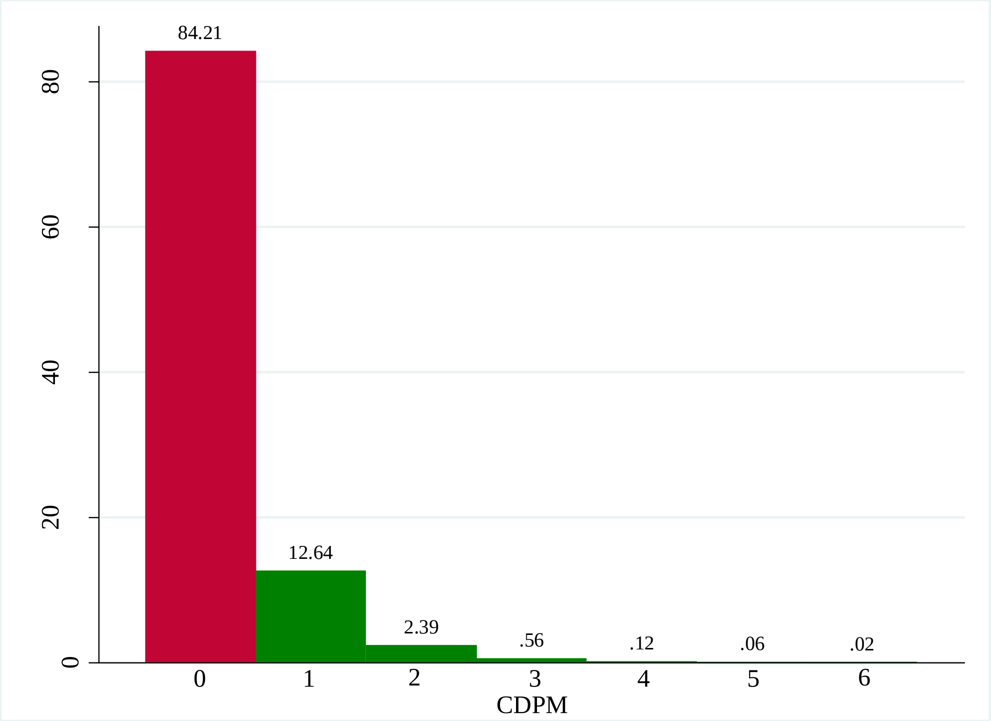

Supplement: Attachment — Submitted filename: PONE-D-24-22195_reviewer.pdf [file pone.0318787.s001.pdf]
